# Solid Propellant Formulations: A Review of Recent Progress and Utilized Components

**DOI:** 10.3390/ma14216657

**Published:** 2021-11-04

**Authors:** Kinga Lysien, Agnieszka Stolarczyk, Tomasz Jarosz

**Affiliations:** 1 Faculty of Chemistry, Silesian University of Technology, 44-100 Gliwice, Poland; kinglys745@student.polsl.pl; 2 Department of Physical Chemistry and Technology of Polymers, Silesian University of Technology, 44-100 Gliwice, Poland; agnieszka.stolarczyk@polsl.pl

**Keywords:** propellant, solid propellant, binder, GAP, energetic material, green

## Abstract

The latest developments in solid propellants and their components are summarized. Particular attention is given to emerging energetic binders and novel, ‘green’ oxidizing agents and their use in propellant formulations. A brief overview of the latest reports on fuel additives is included. Finally, a summary of the state of the art and challenges in its development are speculated on.

## 1. Introduction

Solid propellants have found a wide range of applications, both military and civil in nature. By far their most common use is in rocket engines, such as in the case of sounding rockets used for observation and launch vehicles used for placing satellites in orbit. Besides that, solid propellants also have many applications in civil engineering. Their ability to produce a huge amount of gaseous products is used in air bags. Additionally, as they are substances burning at high temperatures, they are used when destruction of hazardous biological and chemical agents is needed [1].

Depending on their chemical composition, solid propellants are typically classified as:

- Single-base (SB) propellants that contain cellulose nitrate (“nitrocellulose”, NC);
- Double-base (DB) propellants that contain NC and either propane-1,2,3-triyl trinitrate (“nitroglycerin”, NG) or its mixture with other nitric acid esters, such as ethane-1,2-diyl dinitrate (“nitroglycol”, EGDN);
- Triple-base (TB) propellants that are essentially DB propellants supplemented with 1-nitroguanidine;
- Composite propellants (CPs) that contain an oxidising solid (e.g., ammonium perchlorate) and a binder that also acts as a fuel [2].

Apart from the abovementioned components present in the case of the four main solid propellant classes, particular propellant formulations typically also contain a variety of additives, such as preservatives, combustion modifiers, catalysts, curing agents, high explosives, auxiliary fuels, plasticizing agents, and others [3,4]. It should be noted that in some cases, the lines between the individual propellant classes are gradually becoming blurred, as exemplified by the modification of DB propellant formulations with Al powders and 1,3,5,7-Tetranitro-1,3,5,7-tetrazocane (HMX) [5]. 

In terms of their scale of application, SB propellants have long since been replaced by DB propellants as the most commonly used type of solid propellants. In turn, although TB propellants have found some applications, these remain rather niche, such as their application in large-caliber ammunition [6]. Composite propellants, in turn, are becoming increasingly more popular due to the extensive research effort that has been dedicated to their development over recent years [7,8,9,10].

In this work, we have aimed at summarizing the most relevant recent developments in the field of solid propellant formulations. Although the majority of recent reports focus on CPs and their modification. This is exemplified by reports of the use of new energetic binders and novel, ‘green’ oxidizing agents (prospective replacements for the environmentally harmful ammonium perchlorate)), a number of worthwhile efforts are also being undertaken in regards to other types of propellants. This is well exemplified by the attempt to replace the highly problematic liquid nitric acid esters (i.e., NG and EGDN) in double-base propellant formulations with other energetic compounds [11].

## 2. Progress in Composite Propellant (CP) Formulations

### 2.1. Use of New Polymer Binders

**Figure 1:**
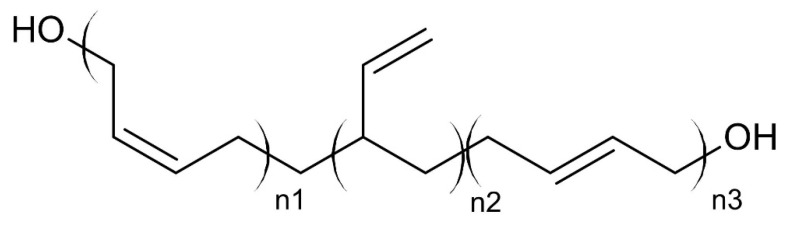
Chemical structure of hydroxyl terminated polybutadiene (HTPB).

Currently, hydroxyl-terminated polybutadiene (HTPB) (Figure 1) is the most commonly used binder in CP formulations, due to its favorable mechanical properties, good adhesion to both hydrophilic and hydrophobic materials, and high heat of combustion [12,13]. HTPB is also reported as resistant to aging, having high oxidative and hydrolytic stability, while allowing for a high degree of loading with solids (up to 90% by weight) [14,15].

Some basic properties of hydroxyl-terminated polybutadiene were listed in Table 1.

**Figure 2:**
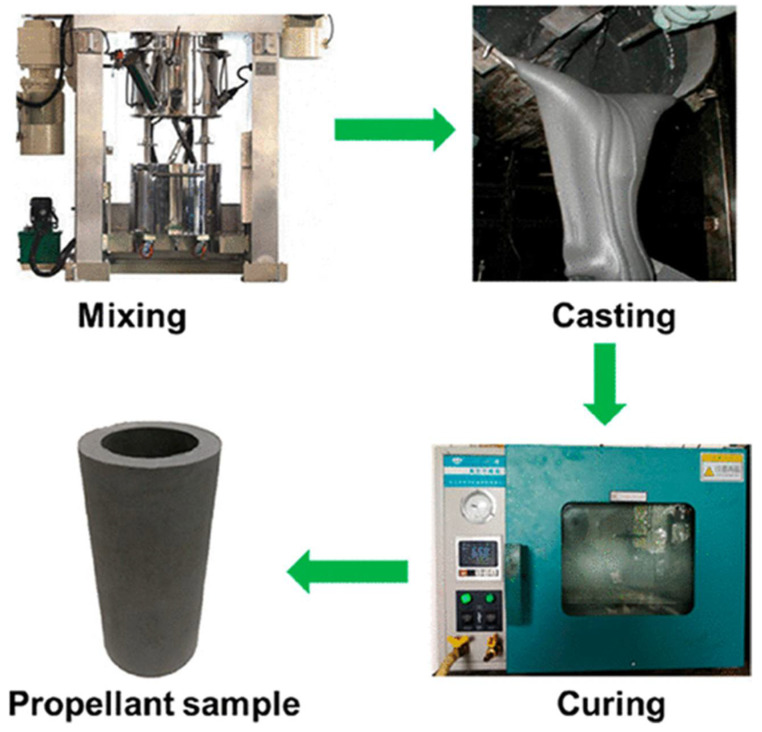
Manufacturing process of HTPB propellant. Reprinted (adapted) with permission from [17]. Copyright 2021, American Chemical Society.

As shown in (Figure 1), non-functionalised HTPB only consists of a hydrocarbon backbone and it exhibits no energetic properties and consequently limits the maximum specific impulse values that can be achieved for formulations containing this binder. Nevertheless, the popularity of HTPB as a binder for propellants stems from its ease of processing (Figure 2). 

That is the reason why new polymeric binders with potential use in rocket propellants are being extensively investigated. Such new materials are varied and even though many HTPB copolymers are reported, most binders for propellants typically are based on different polymers and repeat units, such as glycidyl azide polymer (GAP), 3,3-bisazidomethyl oxetane (BAMO), 3-azidomethyl-3-methyl oxetane (AMMO), as well as their polymers and copolymers. The fundamental properties of some polymeric binders are listed in Table 2.

GAP exhibits a set of properties desirable of an energetic binder for use in solid propellant formulations. At room temperature, it is a highly viscous liquid, making it easy to produce suspensions of solid particles (e.g., metallic fuels, oxidizing agents). GAP exhibits a high enthalpy of formation (957 kJ/kg) due to the presence of an azide group in the structure of the macromolecule [13,24,25]. Another novel energetic binder is the polymer of 3,3′-bis(azidomethyl)oxetane (PBAMO). In the case of PBAMO, both its density and heat of formation are higher than the respective values for GAP, but on the other hand, PBAMO exhibits poor mechanical properties (i.e., higher glass transition temperature) [20,26]. To overcome this problem, a lot of copolymers are synthesized (e.g., DFAMO/BAMO copolymer) [27]. 

**Figure 3:**
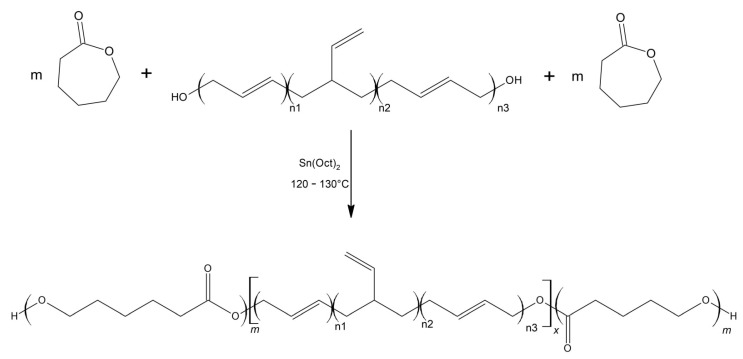
Synthesis of the hydroxyl terminated block copolymer (HTBCP).

In the aspect of new HTPB derivatives, copolymers of HTPB with ε-caprolactone have recently been reported, produced by HTPB-initiated ring opening polymerization of ε-caprolactone (Figure 3) [28]. The ε-caprolactone content was varied among the produced copolymers, revealing that polymers containing 25% by weight of ε-caprolactone units showed the most favorable properties (good viscosity, adequate hydroxyl value, miscibility with nitroglycerine as a plasticizer). Additionally, it provides proper strain capabilities, enables high solid loading and its glass transition temperature remains low (T_g_ = −74 °C). Referring to their results, it is predicted to be possible to obtain a significantly higher specific impulses using such a binders (I_sp_ = 263,6 s), as compared to the traditional HTPB-based propellants (I_sp_ = 260,2 s), as calculated using NASA CEC software. 

This copolymer has also been investigated alongside NG (acting as a plasticizer), in a series of composite propellant formulations [29] using analogous HTPB/DOA formulations for comparison (Table A1).

According to the study, an 12–16% increase of the calorimetric value and 4.4–5% gain in the density is observed for the HTBCP25/NG formulations. Theoretical performance of the formulations was calculated by using NASA CEC-71 code. The obtained results predict that HTBCP25/NG formulations will outperform others, when specific impulse values, characteristic velocity values, flame temperature, and burning rates are considered.

Energetic thermoplastic elastomers (ETPEs) are a big group of compounds, with a good chance to replace the traditional binders. ETPEs can be reused, recycled, and recovered and it has contributed to their prevalence among energy materials. In recent years, much work has focused on the synthesis of poly(3,3-bisazidomethyl oxetane/3-azidomethyl3-methyl oxetane) (P(BAMO/AMMO)), due to its good properties: proper heat formation, low glass transition temperature and low sensitivity to stimuli. PAMMO is usually obtained by the living cationic polymerization of AMMO, which is known for its very high mechanical sensitivity [30,31,32,33,34,35,36,37].

**Figure 4:**
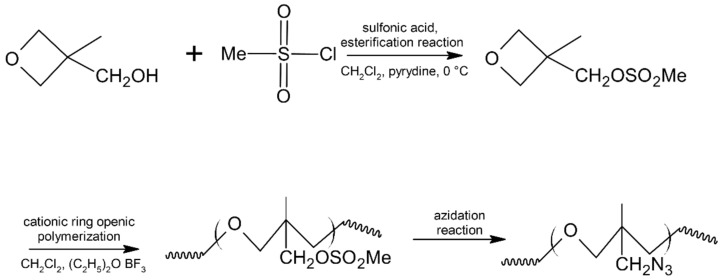
Synthesis of poly(3-mesyloxymethyl-3-methyl oxetane) (PAMMO). Reprinted (adapted) with permission from [38]. Copyright 2021, John Wiley and Sons.

Wang et al. [38] proposed a quicker and safer way to synthesize poly(3-mesyloxymethyl-3-methyl oxetane) (PAMMO) through the cationic ring opening polymerization of MMMO and azidation of obtained PMMMO, as the second stage. Two ways of azidation were investigated: homogenous method and phase-transfer catalyst. In Figure 4, the general synthesis of PAMMO is presented, with MMMO and PMMMO synthesis included.

Results show that phase transfer catalysis method is more efficient than homogenous method (it took 18 h to gain 100% and for homogenous method it took much longer—42 h). The study also reports the thermal decomposition performance of PAMMO by a TG/FTIR/MS analysis. A two stage thermal decomposition can be observed. During the first stage, azide groups are decomposed and gaseous products such as N_2_, HCN, and NH_3_ occurred. Polyether backbone decomposes in the second stage—with NO, NO2, CH2O, and other C-H gases’ release. 

Zhang et.al [39] prepared a BAMO-AMMO alternative block (BAAB)-based propellant, with 80% of the solid content. In this study, a BAAB was used as an ETPE and the final formulation was optimized by making the energy calculations. Theoretical predictions yielded the following: the higher content of RDX and Al, the higher the specific impulse; higher Al content leads to reduction in the oxygen balance coefficient. The formulation predicted to ensure high specific impulse (275.45 s) and appropriate oxygen balance coefficient (0.5) is listed in Table A2.

Density, heat of explosion, glass transition temperature, and mechanical properties were also measured and investigated. Obtained data were better as compared to the similar propellant formulation (e.g., obtained density was 1.8102 g cm^−3^ and for reference sample was 1.7814 g cm^−3^).

**Figure 5:**
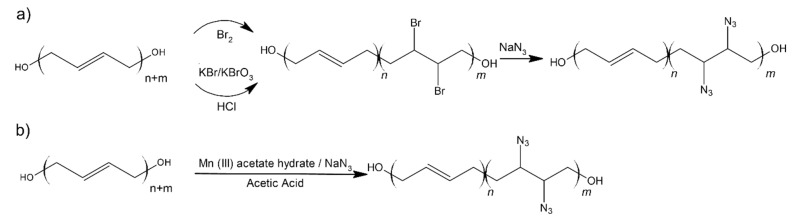
Synthesis of azidated hydroxyl-terminated polybutadiene (HTPB): (**a**) two-step method; (**b**) one-step method.

Pant et al. [15] reported a method for obtaining a functionalized HTPB with azide groups, using two methods (Figure 5).

In the two-step synthesis, the first step involves bromination reaction, followed by azidation. Single step synthesis was based on the method proposed by Fristad et al. [40] and is much easier and faster to carry out. Despite that, the azide groups content is lower, as compared to the product of the two-step synthesis. Moreover, separation of the product was also a problematic issue. 

Additionally, probes with different azido groups content were prepared (10%, 15%, 20%). The study reports that when the azide groups increase, the viscosity and glass temperature also increase. It turned out that the best candidate for further rocket propellant application is a binder with 10% content of azido groups, because glass temperature and viscosity were in the scope of parameters, which gives propellant its practical usage.

### 2.2. Novel Oxidizing Agents

Ammonium perchlorate (NH_4_ClO_4_, AP) is commonly used oxidizing agent for solid rocket propellant. Combustion of fuels, with AP as an oxidising agent, in rocket engines leads to release of hazardous substances, which affect human health and also have bad influence on the environment. Recently, many studies have focused on obtaining new oxidizing agents, by synthesis of novel compounds or modification of known compounds. The aim was to obtain compounds that meet the current requirements for solid rocket fuels (non-polluting, characterized by a good oxygen balance and providing high specific impulse) [41,42,43].

Three different novel oxidizing agents were investigated by Abd-Elghany et al. [44]: Bis(2,2,2-trinitroethyl)oxalate (BTNEOx) [45], 2,2,2-Trinitroethyl-nitrocarbamate (TNENC) [46], and 2,2,2-Trinitroethyl-formate (TNEF) [47]. Obtained results were compared to the traditional AP/HTPB rocket propellant formulation. The performance of the HTPB based propellant formulations with different content of high energy density oxidizing agents was reported. For each ratio, the highest value of specific impulse was predicted (EXPLO5 software) for TNEF, so it was chosen for further investigation and for comparison with AP/HTPB binder (Table 3). The comparison included parameters such as specific impulse (Is), characteristic exhaust velocity (C*), thrust velocity (Cf), temperature at the nozzle exit (Te), and mole of the gaseous products (mol·g).

Higher characteristic exhaust velocity also contributes to the better overall combustion properties. TNEF/HTPB measured burning rate was 14% higher than AP/HTPB (12.11 mm·s^−1^ for TNEF/HTPB and 10.64 mm·s^−1^ for AP/HTPB). 

TNEF was also investigated theoretically against AND in a propellant formulation utilizing GAP as the binder [48]. 

Performance parameters of the prepared samples were predicted using EXPLO5 V_6.03 software, suggesting that the TNEF/GAP formulation will show better performance than the AND/GAP formulation, both in terms of specific impulse (Is = 250.1 s and Is = 202.4 s respectively) and characteristic exhaust velocity (C* = 1408 m s^−1^ and C* = 1243 m s^−1^ respectively). TNEF is not only expected to provide high performance, but also is well miscible with GAP, forming a homogeneous mixture. In the case of ADN/GAP propellant, the aggregation of irregularly shaped ADN crystals and their coating by the polymeric matrix occurred, which may result in unstable and uncontrolled burning of the propellant. In summary, TNEF outperforms the traditional AP/HTPB binder and is a promising novel oxidizing agent to which further research should be devoted to increase the scale of production and explore its practical application in solid rocket propellant formulations. 

**Figure 6:**
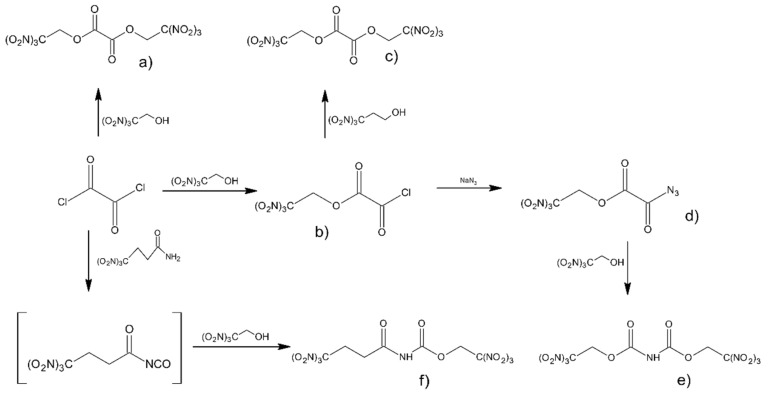
Synthesis of the polynitro compounds.

Klapötke et al. [45] prepared a series of synthesis of new potential high-energy density oxidizing agents. Compounds were synthesized in the reactions of 2,2,2-trinitroethanol (TNE) and 3,3,3-trinitropropanol with oxalyl chloride and hydrazide (Figure 6).

**Figure 7:**
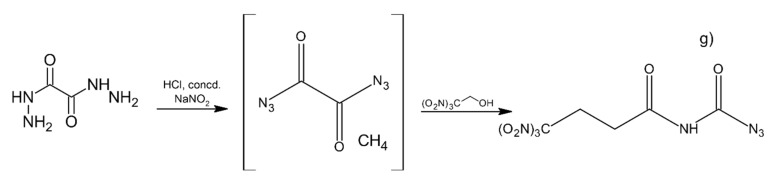
Synthesis of 2,2,2-trinitroethylcarbamate carbonyl azide.

Series of chemical reactions led to formation of bis(2,2,2-trinitroethyl) oxalate (a) 2,2,2-trinitroethyl chloro(oxo)acetate (b), 2,2,2-trinitroethyl (3,3,3-trinitropropyl) oxalate (c), 2,2,2-trinitroethyl azido(oxo)acetate (d), the diester bis(2,2,2-trinitroethyl) imidodicarboxylate (e) and finally 2,2,2-trinitroethyl N-(4,4,4-trinitrobutanoyl)-carbamate (f). 2,2,2-trinitroethylcarbamate carbonyl azide (g) is formed in the reaction of oxalyl diazide with TNE (Figure 7).

For the synthesized compounds, a high decomposition temperature (183–186 °C) was observed. Sensitivity measurements were also a subject of this study. Among those compounds, one was found to be extremely sensitive to impact and fraction stimuli and great caution should be taken while working with such a compound. Summarizing measured parameters for all obtained compounds are in the range achievable with the use of traditional oxidizing agents, but the predicted specific impulse value was still below the value calculated for ammonium perchlorate (Is = 256 s, as compared with NH_4_ClO_4_—Is = 262 s). 

**Figure 8:**
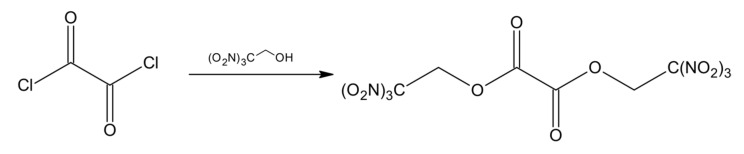
Synthesis of BTNEOx.

Another study [11] investigated the thermal behavior and decomposition characteristics of Bis(2,2,2-trinitroethyl)-oxalate (BTNEOx) (Figure 8) and its formulation with nitrocellulose. In this study an attempt was made, to prove that NC/BTNEOx is a proper replacement for nitroglycerine in smokeless double base propellant. High frame rate recordings were used to prove that during the combustion of propellant, smokeless gases are formed. Further investigation also confirmed that NC/BTNEOx formulations is an interesting replacement for NG, so further research should be undertaken.

One of the most popular research topics in the field of the novel energetic materials are co-crystals—crystalline materials, which consist of two or more components and exhibiting unique sets of properties. It is considered that this relatively new technology improves the mechanical properties of explosives and may also contribute to improved combustion rates and oxygen balance. Moreover, co-crystallization may also contribute to the increase of thermal stability.

Recently, many studies [49,50,51] on development of co-crystals, such as a co-crystal composed of hydrazine 3-nitro-1,2,4-triazol-5-one (HNTO) and ammonium nitrate (AN). This co-crystal was developed in order to overcome some of the drawbacks (i.e., high hygroscopicity and phase transitions of AN and negative oxygen balance of HNTO) of AN and HNTO [49]. A noticeable difference in the morphology and size of the co-crystal and the parent substance crystals was observed (as shown by X-ray diffractograms), despite similar crystallization conditions.

Thermal properties of the raw materials and co-crystal were investigated with a DSC method. The co-crystallization has changed the thermal properties of the obtained material and as a result, the co-crystal does not go phase transformation. The heat of decomposition (1483.15 J/g) was also higher than that of the parent substances (940.20 J/g and 849.79 J/g for AN and HNTO respectively).

High mechanical sensitivity of the HNTO limits its practical application in SRP or explosives. Obtained co-crystal has only few surface defects and voids (possibility of the generation of the hot spots under mechanical stimuli decreases).

**Figure 9:**
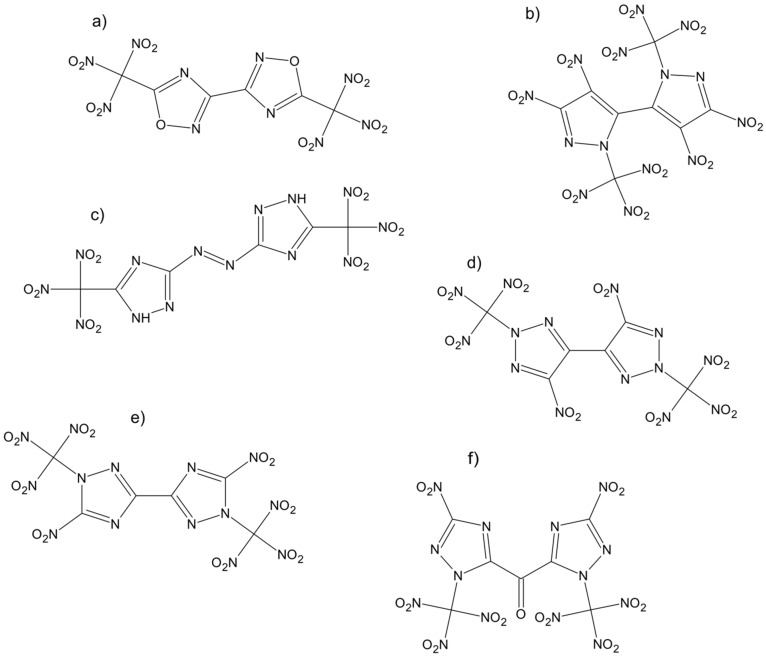
Novel bis-heterocyclic compounds.

Another example of recently obtained novel green oxidizing agents are bis-heterocyclic compounds. When compared to single heterocyclic compounds, bis-heterocyclic oxidizing agents are characterized with better properties, relevant to such compounds (e.g., higher detonation performance and thermal stability). Recently, five novel bis-heterocyclic compounds with potential use as oxidizing agents were synthesized (Figure 9a–f) [45,47,52,53,54,55,56].

In the literature, one more example of bis-heterocyclic compound has been described: Bis(3-nitro-1-(trinitromethyl)-1H-1,2,4-triazol-5-yl)methanone (Figure 9f) [57].

**Figure 10:**
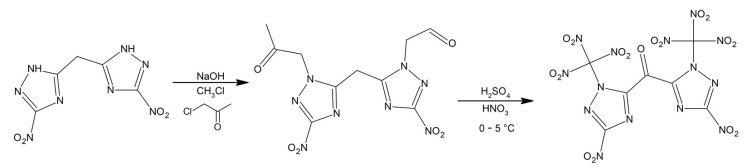
Synthesis of bis(3-nitro-1-(trinitromethyl)-1H-1,2,4-triazol-5-yl)methanone. Reprinted (adapted) with permission from [57]. Copyright 2021, American Chemical Society.

The authors attempted to obtain a compound that would have similar (high) density and good impact sensitivity, while improving the decomposition temperature. The proposed synthesis proceeds in two steps and involves treating bis(3-nitro-1H-1,2,4-triazol-5-yl)methane with a sodium hydroxide and chloroacetone in acetonitrile. The next step is the nitration reaction in the presence of sulfuric and nitric acids (Figure 10).

Characteristic parameters for rocket propellants were calculated using EXPLO5 (version 6.01) software. 

By comparing the data in Table 4, it can be concluded that bis(3-nitro-1-(trinitromethyl)-1H-1,2,4-triazol-5-yl)methanone is a promising candidate for being used as an oxidizing agent in rocket propellant compositions. It is characterized with a high density and a proper, high decomposition temperature. Furthermore, its theoretical specific impulse is higher than AP or ADN.

### 2.3. Effect of the Additives on Propellant Efficiency and Other Properties

Oxidant and binder are the two main components of the solid rocket fuel. Despite these, propellants formulations usually consist of appropriate additives, which influence their overall performance and other properties (e.g., mechanical properties). Commonly used propellant additives include metal fuel, curing agents, burning rate catalysts, etc. [58,59,60,61].

#### 2.3.1. Effects of the Additives on the Mechanical Properties

The purpose of plasticizers as rocket fuel additives is to significantly improve the fuel processing properties [4]. Boshra et al. [62] investigated different composite propellant formulations, which vary in the used plasticizer. Plasticizer they used were as follow: dioctyl adipate (DOA), bis(2-ethylhexyl) azelate (DOZ), dibutyl phthalate (DBP). 

This study reveals that using DOZ and DBP as a plasticizer in GAP-based SRP formulations leads to increasing the viscosity and accelerating the curing reactions. After the test, the separation between fillers and binder occurred, which proves that DOZ is incompatible with GAP. The same phenomena were noticed for DBP. DOA was chosen to be the best plasticizer for GAP matrix: propellant was characterized with a low viscosity and high tensile strength. 

Cross-linking agents are the critical component when it comes to mechanical properties of the propellants. In one study [63], different crosslinking mixtures based on trimethylolpropane (TMP) as a crosslinker and butanediol (BDO) as a chain extender on CSRPs based on hydroxyl-terminated polybutadiene, were studied, with 27 propellant samples being prepared with different weight ratio of TMP to BD and investigated. The effect of CM content (0–0.5%) on propellant properties was also investigated. Moreover, the effect of the CM on CSRPs with different ratio of NCO/OH = 0.7, 0.75, and 0.8 was studied to indicate the proper ratio, that enables the largest possible strain-ability and high strength.

The importance of the NCO/OH ratio in generating the crosslinking and binding between chains of the polymeric matrix was proved: rise in the strength and a reduction in the strain were observed as NCO/OH increases was observed for all the mixtures. The highest value of tensile strength was measured for TMP-BDO (2:1 ratio). It varied from 8.2 kgf/cm^2^ (TMP-BDO 0.15%), 11.2 kgf/cm^2^ (TMP-BDO 0.3%), and 12.1 kgf/cm^2^ (TMP-BDO 0.5%) at NCO/OH = 0.7; 10.1 kgf/cm^2^ (TMP-BDO 0.15%), 12.5 kgf/cm^2^ (TMP-BDO 0.3%), and 14.1 kgf/cm^2^ (TMP-BDO 0.5%) at NCO/OH = 0.75; and finally 11.5 kgf/cm^2^ (TMP-BDO 0.15%), 13.8 kgf/cm^2^ (TMP-BDO 0.3%), and 15.7 kgf/cm^2^ (TMP-BDO 0.5%) at NCO/OH = 0.8. In addition, as the TMP-BDO mixture content increases, the rate in tensile strength also increases and gives the highest strength of kgf/cm^2^ at 0.5% TMP-BDO content, when compared to the other mixtures added. The reason for this is a higher triol (TMP) ratio, which leads to more crosslinks between chains and formation of the three-dimensional polymeric matrix.

#### 2.3.2. Effects of the Additives on the Performance Properties

In another study, 56 nm particles of iron(III) oxide were used as a combustion rate modifier in SRP formulations [64]. AP/HTPB rocket fuel samples with different Al and Fe_2_O_3_ content were prepared and investigated under the rule the more Fe_2_O_3_ the less AP was added.

According to study results, calorific value of the propellants, sensitivity to friction and hardness decreases when the Fe_2_O_3_ content increases. Additionally, the catalytic effect of the Fe_2_O_3_ on the burning rate of propellant was proven. 

An application of [Cu(TNBI)(NH_3_)_2_(H_2_O)] as an energetic complex [65] in solid rocket propellants has been studied. Rocket fuel was based on the AP/HTPB formulation and a complex was used to replace RDX, which is considered as a high explosive compound in the propellant formulation. Different compositions were prepared. Although the study reports that big amount of [Cu(TNBI)(NH_3_)_2_(H_2_O)] decreases the calorific value of the propellant (when compared to HTPB-based samples), authors state that [Cu(TNBI)(NH_3_)_2_(H_2_O)] can be used as a replacement for RDX. On the other hand, addition of complex results in improvement of the hardness and reduces the sensitivity to friction. Higher burning rates were obtained for samples with [Cu(TNBI)(NH_3_)_2_(H_2_O)], so it may also be used as a burning rate modifier and studies on this aspect will be progressed. 

The influence of additions of Ti and Mg on the thermal properties and the combustion characteristics of HTPB-based solid rocket propellants was also investigated [52]. 

Study reports that addition of Mg/Ti particles lead to decrease of the energy value (because it has lower energy content than boron). On the other hand, the Ti content increase has influence on reduction of the ignition delay time. According to the literature, Ti content of 15–20% by weight of boron content provides the optimal value of the ignition delay time—much lower when compared to B-HTPB based samples. However, addition of Mg particles was characterized with a higher ignition delay time. Differences between ignition value time of samples may be connected with thermal conductivity of the particles [66,67].

The thermal behavior and thermal properties of promising energetic material dihydroxylammonium 5,50-bistetrazole-1,10-diolate (TKX-50) were investigated with different techniques [68]. There are many works dedicated to the investigation of different heterocyclic systems (imidazole, triazole, pyrozole, and tetrazole) [69,70,71,72,73]. Of these heterocyclic systems, the tetrazole moiety has the highest nitrogen atom content, which results in high heat of formation and high energetic performance. Recently, a big amount of nitrogen-rich, tetrazole derivatives were reported [74,75,76,77,78,79]. Although among those systems TKX-50 appears to be the most promising, with its synthesis procedure being reported [80], much work needs to be done to confirm the practical applicability of this compound. TKX-50 is characterized by excellent heat-resistance properties: its critical temperature of thermal explosion is 533.39 K [74]. It was shown that TKX-50 decomposes with the formation of N_2_, H_2_O, NH_3_, N_2_O, and NO.

HTPB-based propellant formulations were made with a iron hydroborate (BH-Fe) as a burning rate modifier were studied [81,82]. The samples had the same composition but differed in mass fraction of BH-Fe (Table A3 and Table A4). Propellant formulations without BH-Fe were used as a reference. The work focuses on describing the effect of BH-Fe on combustion properties (burning rate and pressure exponent).

BET and SEM analysis showed that microstructure of the tested BH-Fe particles has irregular shape and tends to disperse. In addition, no cold cohesion was observed (for the microsized sample). This phenomenon appears during storage, handling, and manufacture (at room temp.) and leads to microsized clusters reducing the specific surface.

Density of propellant formulations which contain aluminum particles is larger than those which contain BH-Fe as an additive. It may be related to the modest porosity of BH-Fe powders during the manufacture of propellant formulation.

When it comes to combustion properties (burning rate and pressure exponent), increasing the mass fraction of BH-Fe leads to increase the burning rate (12% higher burning rate for 3% mass fraction of the BH-Fe replaced Al powder) and experimentally-determined specific impulse.

**Figure 11:**
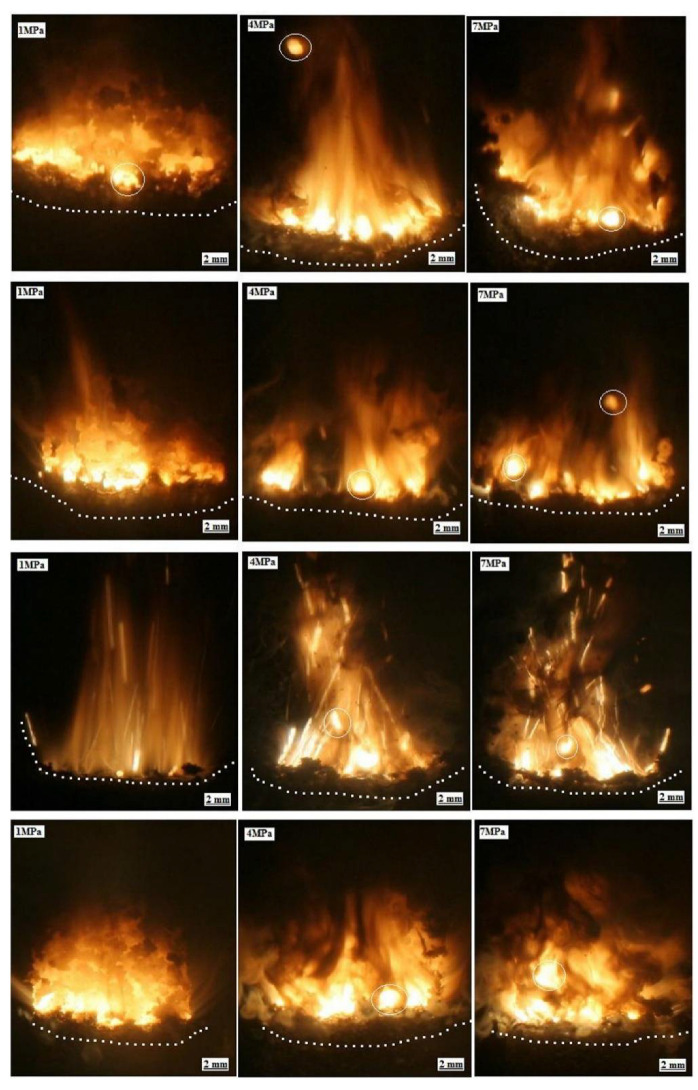
Combustion flame overview propellants with different mass fraction of HIC at pressures 1, 4, and 7 MPa. Top is sample BHF-1, second and third lines are samples BHF-2 and BHF-3, bottom is BHF-4. Reprinted with permission of Elsevier from [82].

Furthermore, the results obtained indicate that although burning rate depends on the mass fraction of BH-Fe, the pressure exponent does not change much. Figure 11.

Effect of Al addition to composite-modified double based propellants on the heat of explosion, burning rate and the combustion properties was reported [5]. Seven formulations with different Al/HMX content were prepared by a slurry-cast method. Heats of explosion, burning rates, combustion parameters and flame characterization were investigated. 

The minimum free energy method (NASA-CEA) was used to calculate the theoretical value of the specific impulse. Study reports that stochiometric coefficient of propellant decreases with the Al content growth. On the other hand, specific impulse increases with the addition of Al (but the increase gradually slows). Heat of explosion increases when the content of Al/HMX increases. With the increase of Al/HMX content, the energy performance also increases—this growth is limited by oxygen balance (which is reduced). At some point (Al/HMX more than 7/30) burning rate is reduced, because of the heat lost. This is caused by the ‘thermal sink’ effect of Al. Furthermore, addition of Al leads to formation of the burning droplets on the propellant surface, which caused to brighter individual flames.

Spinel compounds are further example of additives for composite solid propellants, which can act as ballistic modifiers. Recently, a synthesis of such a compound was reported [83]. Authors aimed to obtain CuCr_2_O_4_ with a excess of CuO. Previously published works have shown the possibility of synthesizing CuCr_2_O_4_ through ceramic method (CeCC), co-precipitation route (CpCC) [84], and Pechini method. CuCr_2_O_4_ with an excess of CuO was obtained by a decomposition of the sodium alginate mixed metal complex. Crystalline structure was analyzed using X-ray diffraction (XRD) spectroscopy. Observation of tetragonal peaks (101) of CuCr_2_O_4_ confirmed the spinel structure. Additionally, further observations confirmed CuO phase occurrence. 

The resulting compound was tested in a typical solid propellant formulation (AP/HTPB) in order to determine its burning rate. Additionally, results were compared with SRP formulation without ballistic modifier (Table 5).

As it can be seen in the Table 4, addition of CuCr_2_O_4_ resulted in increase of burning rate of propellant in 43%. The reason for the increased burning rate is the appearance of the additional catalytic areas, which accelerate the oxidation of propellant components [85,86].

## 3. Summary

In recent years, extensive work has been performed on novel energetic binders, as well as other components of propellant formulations. These efforts have largely been focused on making propellants more ‘green’, by replacing the most environmentally-harmful components of their formulations. The replacement of ammonium perchlorate is of particular research interest and numerous energetic systems have been proposed as alternatives to this compound, particularly nitrogen-rich organic cyclic species and their salts.

In terms of the evolution of binders used in propellant formulations, recent years have brought a shift away from the ‘tried and true’ use of HTPB, in favour of more energetic polymers, such as GAP and PBAMO. This stems from the fact that such binders can actually improve the performance of propellants rather than only serving as an organic fuel and thermal ballast, as in the case of HTPB.

The use of additives has also been shown to be an important aspect of developing rocket propellant formulations as they can greatly affect the final performance of a propellant. Nevertheless, it should be remembered that if the fundamental components of a propellant formulation are not optimized, no amount of additives will resolve the arising performance issues.

In terms of practical applications, the greatest challenge for the development of new propellant formulations is not the lack of high-performance components, but the effective cost of a unit amount of propellant. Although cost may be disregarded in some applications, in most cases, the use of components that are produced through a sequence of sophisticated chemical transformations will result in a prohibitive cost of the propellant, greatly limiting its prospective applications.

## Figures and Tables

**Table 1:** Fundamental properties of HTPB binder [16].

Number Average Molecular Weight (Da)	5210
Dispersity	2.53
Viscosity (cp) (T = 27 °C)	2320
Hydroxyl Value (mg KOH/g)	42
Microstructure (%) (cis, Trans, Vinyl) ^a^	29, 64, 7

^a^—it was calculated from ^1^H-NMR spectra.

**Table 2:** Fundamental properties of typical binders.

Polymer	Density (g/cm^3^)	ΔHf (kJ/mol)	OB %	Tg (°C)	Lit.
HTPB	0.90–1.50 ^a^	261–1290 ^a^	−65	−75	[16,18]
GAP	1.30	117	−45	−45	[19,20]
PBAMO	1.30	413	−45	−39	[20,21]
PAMMO	1.17	179	−35	−170	[20,22]
PolyNIMMO	1.26	−335	−25	−114	[20,22]
PolyGLYN	1.39	−285	−65	−61	[22,23]

^a^—depends on the source (as well as compound composition).

**Table 3:** Comparison between performance of AP/HTPB and TENF/HTPB propellant formulations [47].

Formulation	AP/HTPB	TNEF/HTPB
Is (s)	245.9	251.2
C* (m·s^−1^)	1484.4	1532.6
Cf	1.62	1.61
Te (K)	1360.5	1331.8
Molg (mol·kg^−1^)	39.77	41.13

**Table 4:** Comparison of the properties of the described compound with AP and AND [57].

Compound	Bis(3-nitro-1-(trinitromethyl)-1H-1,2,4-triazol-5-yl)methanone	AP	ADN
ρ (g/cm^3^)	1.95/1.93	1.95	1.81
V_DET_ (m/s)	8252	6368	7860
T_dec_ (°C)	164	>200	159
IS (J)	9	15	3–5
I_sp_ (s)	219	157	202

**Table 5:** Comparison between burning rates of propellant samples [83].

Pressure (MPa)	Burning Rate WOMO ^a^ (mm/s)	Burning Rate CURCO ^b^ (mm/s)
4.90	6.82	9.73
8.83	8.36	12.00

^a^—propellant without metal oxide. ^b^—propellant with CuCr_2_O_4_.

## Data Availability

Not applicable.

## Appendix A

**Table A1:** Compositions of propellants [29].

	HTPB	HTBCP25	DOA	NG	Others ^a^	AP ^b^	Al	RDX
I	10	-	5	-	1	64	20	0
II	-	10	-	5	1	64	20	0
III	10	-	5	-	1	59	20	5
IV	-	10	-	5	1	59	20	5
V	10	-	5	-	1	54	20	10
VI	-	10	-	5	1	54	20	10
VII	10	-	5	-	1	49	20	15
VIII	-	10	-	5	1	49	20	15

^a^—crosslinker (trimethylol propane); chain extender (n-butanediol); curing agent (IPDI). ^b^—total AP (300 µm, 60 µm).

**Table A2:** Propellant formulation [39].

Compound	Mass Fraction (%)
BAAB ETPE	15
AP	40
RDX	18.5
Al	18
BuNENA	5
Carbon black	1.4
PbCO_3_	2.1

**Table A3:** Composition of propellant samples used in study [81].

Binder	HTPB
Plasticizer	di-2-etylhexyl sebacate (DES)
Curing agent	2,4-toluene diisocyanate (TDI)
Cross-linking agent	isophthaloyl-bis-(2-methylaziridine) (HX-752)
Bonding agent	bi-(2-methy-1-aziridinyl) phosphine oxide (MAPO)
Metallic fuel	Al powder (5µm)
Oxidising agent	AP
Burning rate modifier	catocene (GFP); chromite copper (CC)

**Table A4:** Mass fraction of formulation’s compounds [81].

Samples	HTPB (%)	AP (%)	Al (%)	GFP (%)	CC (%)	BH-Fe (%)	Add. (%)
CSP-1	9.5	65	18	4	1.5	0	2
CSP-2	9.5	65	17	4	1.5	1	2
CSP-3	9.5	65	15	4	1.5	3	2

CSP-1—reference sample.
